# Evaluation on curative effects of percutaneous endoscopic lumbar discectomy via a transforaminal approach versus an interlaminar approach for patients with lumbar disc herniation: A protocol for systematic review and meta-analysis

**DOI:** 10.1097/MD.0000000000027089

**Published:** 2021-10-01

**Authors:** Rui Li, Bo Chen, Weiwen Shen, Qing Wang

**Affiliations:** a Department of Spine Surgery, Suizhou Hospital, Hubei University of Medicine, Suizhou, Hubei, China; b Department of Radiology, East Hospital, Tongji University School of Medicine, Shanghai, China.

**Keywords:** curative effects, disc herniation, lumbar, meta-analyse, percutaneous endoscopic interlaminar discectomy, percutaneous endoscopic transforaminal discectomy

## Abstract

**Background:**

The present meta-analysis aims to conduct a systematic comparative study of the curative effects of Percutaneous Endoscopic Transforaminal Discectomy (PETD) and Percutaneous Endoscopic Interlaminar Discectomy (PEID) when used to treat Lumbar Disc Herniation (LDH).

**Methods:**

The following online databases will be searched to find articles related to treating of LDH using PETD and PEID, namely PubMed, EMBASE, Cochrane databases, China National Knowledge Infrastructure, WanFang Database, and Web of Science. The search will include all articles published from inception until August 2021. Articles will be included according to the inclusion criteria. Two authors will independently screen and assess the quality of each study. RevMan (version 5.3) will be adopted to complete data analysis and evaluate the statistical significance of each operating technique in various outcomes.

**Results:**

The present meta-analysis will evaluate the curative effects of using PETD and PEID to treat LDH patients.

**Conclusion:**

Compare the clinical efficiency of PETD and PEID as therapy for LDH with postoperative hypoesthesia, complications in surgical site wounds, nerve root injury, transfer to open surgery, recurrence, partial decompression, and other complications. The comparative analysis will help determine the difference between the 2 surgical methods of PETD and PEID for treating LDH.

**OSF registration number:**

August 2, 2021.osf.io/hr964. (https://osf.io/hr964/)

## 1 Introduction

Lumbar disc herniation (LDH) is a highly prevalent condition, primarily due to different sections of the lumbar intervertebral disc having varying levels of degenerative changes. During exertion of high external force, there is a rupturing of the fibrous annulus of the intervertebral disc. As a result, there is a protruding of nucleus pulposus tissue from the rupture (prolapse) in the back or in the spinal canal, which causes irritation as the adjacent spinal nerve roots compresses, giving rise to lumbar pain, a numbing feeling, and discomfort in the lower limbs, and a series of clinical symptoms.^[1–3]^ At present, surgically treating lumbar disc herniation exhibits positive outcomes. Surgeons adopt a variety of surgical methods depending on the case, which includes both open surgery and various minimally invasive surgery.^[4,5]^ The recent advancement of Minimally Invasive Spinal Surgery technology has allowed surgeons to adopt Percutaneous Endoscopic Lumbar Discectomy as a routine operation for LDH. Percutaneous Endoscopic Transforaminal Discectomy (PETD) and Percutaneous Endoscopic Interlaminar Discectomy (PEID) are the 2 primary approaches of Minimally Invasive Spinal Surgery. Different clinical surgical approaches have distinctive characteristics and indications. When compared with traditional open surgery, intervertebral foraminal nucleus pulposus excision has the benefits of short preoperative preparation time, use of local aesthesia, minimally invasive, less intraoperative bleeding, low infection rate, and low risk of nerve damage and thrombosis.^[6]^ However, with the increasing popularity and development of intervertebral foraminal surgery, there are many reports on the treatment of lumbar intervertebral disc herniation by percutaneous foraminal endoscopy, both domestically and overseas. However, some scholars believe that PETD reduces the occurrence of complications. Meanwhile, some scholars are convinced that PEID is simpler and can lessen the time taken to perform the surgery as well as the number of intraoperative fluoroscopies. Currently, there is no meta-analysis on the 2 surgical approaches. Therefore, the present meta-analysis will conduct a multifaceted analysis of the PETD and PEID approaches to present comprehensive evidence to determine the best surgical method.

## 2 Materials and methods

### 2.1 Eligibility criteria

#### 2.1.1 Inclusion criteria

Articles that satisfy the following inclusion criteria will be included:

1. Randomized controlled trials (RCT) and cohort studies on using PETD and PEID to treat LDH, both at home and abroad;
2. Patients diagnosed with lumbar disc herniation through physical examination and imaging assessments (computed tomography, magnetic resonance imaging, and X-ray), but have not undergone surgical treatment after 3 months of conservative treatment;
3. Papers in English or Chinese.

#### 2.1.2 Exclusion criteria

Articles that meet the following exclusion criteria will be omitted:

1. Repetitive research, review articles, case reports, biomechanics, and cadaver research, studies with incomplete or unavailable data, and when the original author of a study cannot be contacted;
2. The study includes patients with spinal infections, acute fractures, tumors, deformities, osteoporosis, or rheumatoid arthritis;
3. Research related to multiple segments of intervertebral disc herniation and reoperation.

### 2.2 Search strategy

The present meta-analyse is based on the PRISMA System Evaluation Guidelines.^[7]^ Two researchers will independently search the online databases listed below from its inception to July 20, 2021: PubMed, EMBASE, Cochrane databases, China National Knowledge Infrastructure, WanFang Database, and Web of Science. The following keywords will be used to search the databases: lumbar disc herniation, percutaneous endoscopy, transforaminal approach, and translaminar approach. A free combination of search terms will be used to expand the search scope.

### 2.3 Data extraction and quality assessment

A couple of authors will extract relevant data independently from the selected studies and cross-check them. All disagreements will be arbitrated via discussions or negotiations by including a third party. Primary outcomes include surgical time, intraoperative fluoroscopy, intraoperative blood loss, complications related to the surgical process, visual analogue scale, and Oswestry disability index (the Oswestry disability index).

The Newcastle-Ottawa Scale will be used to evaluate the quality of included cohort studies. The RCT bias risk assessment tool recommended by Cochrane Handbook 5.1.0 will be adopted to evaluate the bias risk of the included RCTs.^[8,9]^

### 2.4 Statistical analysis

The present analysis will use an odds ratio to calculate the dichotomous data. Meanwhile, mean difference with 95% confidence interval will be adopted to represent continuous data. Moreover, the heterogeneity among collective study outcomes will be evaluated using the Cochran *Q* test and the degree of inconsistency (*I*^2^). In the case where *P* > .05 and *I*^2^ < 50%, a fixed-effects model will be used to pool data. Else, a random effects model will be used to pool the data. In the integration results, a *P* < .05 will be considered statistically significant. Standard methodology will be used to assess and treat the publication bias in outcomes. Moreover, the publication bias will be analyzed through funnel plots.

### 2.5 Ethics and dissemination

An ethics approval is not required for this study since the systematic review and meta-analysis uses prepublished data.

## 3 Discussion

Globally, there is an increasing ageing population, combined with the changes in people's lifestyles brought about by social progress, there is a steady increase in the number of lumbar disc herniation patients each year. Percutaneous endoscopic spine surgery is becoming increasingly mature with the development of minimally invasive endoscopic technology.^[10]^ In contrast with conventional standard discectomy and standard small fenestration discectomy, percutaneous intervertebral foraminoscopic surgery exhibits a greater surgical success rate with lower complications. Generally, the efficacy and safety of the PETD and PEID methods are well documented, both locally and internationally. However, clinical research reports still have contrasting opinions on the efficacy of the 2 approaches for treating lumbar disc herniation. The present article will meta-analyze these 2 approaches to help clinicians make evidence-based decisions.

## Author contributions

**Conceptualization:** Rui Li, Bo Chen.

**Data curation:** Rui Li, Bo Chen, Weiwen Shen, Qing Wang.

**Formal analysis:** Rui Li, Bo Chen, Weiwen Shen, Qing Wang.

**Funding acquisition:** Weiwen Shen, Qing Wang.

**Investigation:** Rui Li, Bo Chen.

**Methodology:** Rui Li, Bo Chen, Weiwen Shen.

**Resources:** Rui Li, Bo Chen, Weiwen Shen, Qing Wang.

**Software:** Rui Li, Bo Chen, Qing Wang.

**Supervision:** Weiwen Shen, Qing Wang.

**Validation:** Rui Li, Weiwen Shen.

**Visualization:** Rui Li, Bo Chen.

**Writing – original draft:** Rui Li, Weiwen Shen, Qing Wang.

**Writing – review & editing:** Qing Wang.

## References

[1] Deyo RA Mirza SK. CLINICAL PRACTICE. Herniated lumbar intervertebral disk. N Engl J Med 2016;374:1763–72. 27144851 10.1056/NEJMcp1512658

[2] Benoist M. The natural history of lumbar disc herniation and radiculopathy. Joint Bone Spine 2002;69:155–60. 12027305 10.1016/s1297-319x(02)00385-8

[3] Kaneuchi Y Sekiguchi M Kameda T. Temporal and spatial changes of (-opioid receptors in the brain, spinal cord and dorsal root ganglion in a rat lumbar disc herniation model. Spine (Phila Pa 1976) 2019;44:85–95. 30005035 10.1097/BRS.0000000000002776

[4] Blamoutier A. Surgical discectomy for lumbar disc herniation: surgical techniques. Orthop Traumatol Surg Res 2013;99: 1 suppl: S187–96. 23352565 10.1016/j.otsr.2012.11.005

[5] Tu Z Li YW Wang B. Clinical outcome of full-endoscopic interlaminar discectomy for single-level lumbar disc herniation: a minimum of 5-year follow-up. Pain Physician 2017;20:E425–e430. 28339442

[6] Minamide A Yoshida M Yamada H. Endoscope-assisted spinal decompression surgery for lumbar spinal stenosis. J Neurosurg Spine 2013;19:664–71. 24093466 10.3171/2013.8.SPINE13125

[7] Moher D Liberati A Tetzlaff J. Preferred reporting items for systematic reviews and meta-analyses: the PRISMA statement. PLoS Med 2009;6:e1000097. 19621072 10.1371/journal.pmed.1000097 PMC2707599

[8] Stang A. Critical evaluation of the Newcastle-Ottawa scale for the assessment of the quality of nonrandomized studies in meta-analyses. Eur J Epidemiol 2010;25:603–5. 20652370 10.1007/s10654-010-9491-z

[9] Hopp L. Risk of bias reporting in Cochrane systematic reviews. Int J Nurs Pract 2015;21:683–6. 24621329 10.1111/ijn.12252

[10] Ahn Y Lee SH Park WM. Percutaneous endoscopic lumbar discectomy for recurrent disc herniation: surgical technique, outcome, and prognostic factors of 43 consecutive cases. Spine (Phila Pa 1976) 2004;29:E326–32. 15303041 10.1097/01.brs.0000134591.32462.98

